# Novel diagnostic approaches and therapeutic management of mucormycosis: insights from a retrospective monocentric cohort study

**DOI:** 10.3389/fmed.2026.1715663

**Published:** 2026-02-04

**Authors:** Othman Lemhamdi, Evelyne Willems, Frédéric Baron, Bernard De Prijck, Jo Caers, Antoine Bouquegneau, Julien Guiot, François Cousin, Alexandre Jadoul, Florence Rogister, Frederic Frippiat, Marie-Pierre Hayette, Adrien De Voeght

**Affiliations:** 1 Division of Hematology, Department of Medicine, University Hospital of Liège, Liège, Belgium; 2 Division of Nephrology-Dialysis-Transplantation, Department of Medicine, University Hospital of Liège, Liège, Belgium; 3 Division of Pneumology, Department of Medicine, University Hospital of Liège, Liège, Belgium; 4 Division of Radiology, Department of Medicine, University Hospital of Liège, Liège, Belgium; 5 Division of Nuclear Medicine and Oncological Imaging, Department of Medicine, University Hospital of Liège, Liège, Belgium; 6 Division of Otorhinolaryngology, Department of Medicine, University Hospital of Liège, Liège, Belgium; 7 Division of Infectiology, Department of Medicine, University Hospital of Liège, Liège, Belgium; 8 Division of Microbiology, Department of Medicine, National Reference Centre for Mycosis, University Hospital of Liège, Liège, Belgium

**Keywords:** antifungal therapy, hematological malignancies, invasive fungal infections, Mucorales, mucormycosis

## Abstract

**Background:**

Mucormycosis is a rare, opportunistic fungal infection with high mortality rates, predominantly affecting immunocompromised patients. Its management is challenging due to diagnostic complexity and limited therapeutic options.

**Methods:**

This retrospective cohort study analyzed 21 cases of mucormycosis diagnosed at the University Hospital of Liège between July 2018 and August 2023. Clinical, microbiological, and radiological data were evaluated to identify risk factors, treatment modalities, and outcomes.

**Results:**

Hematological malignancies (82%) and immunosuppressive therapy (53%) were the most common risk factors. Pulmonary involvement occurred in 94% of cases. Liposomal Amphotericin B was the most frequently used antifungal therapy, and surgery improved outcomes in 50% of cases when it was performed. Despite these measures, the mortality rate on day 84 remained significant at 35%.

**Conclusion:**

This study underscores the challenges of managing mucormycosis in immunocompromised patients. The high mortality rate emphasizes the importance of early diagnosis, tailored antifungal therapy, and standardized treatment protocols to improve survival outcomes.

## Introduction

Mucormycosis, formerly known as zygomycosis, is an acute opportunistic infection first documented in 1855 by Kurchenmeister. The causative fungi belong to the phylum Glomeromycota and are considered ubiquitous, as they are commonly present in the environment to which humans are routinely exposed (1).

Mucormycosis is a life-threatening invasive fungal infection, characterized by a high mortality rate of up to 61% (2) and limited therapeutic options, often leading to death within a brief period. Mucormycosis often occurs among immunocompromised patients essentially suffering from hematological malignancies, whether they have undergone hematopoietic stem cell transplantation (HSCT) or not (2).

In Belgium, an epidemiological study conducted between 2000 and 2009 revealed a rising incidence of invasive mucormycosis, with the annual rate increasing from 0.019 per 10,000 patient-days in 2000 to 0.148 per 10,000 patient-days in 2009. This study highlighted the rarity of this infectious disease. The prognosis for such infections remains poor, as evidenced by the study’s reported mortality rate of 65% (3).

An increase in incidence has been observed over the past two decades, coinciding with the emergence of various risk factors, diabetes described as the most prevalent one (4). However, recent European studies indicate that the epidemiology of mucormycosis has evolved due to advances in diagnostic tools, an expanding at-risk population, and the use of azoles for *Aspergillus* prophylaxis among patients with hematological malignancies, representing now the most prominent underlying condition (4, 5). Additional factors, such as the administration of high dose corticosteroids in patients with Graft Versus Host Disease (GVHD), have further increased susceptibility to mucormycosis (2). Also, other epidemiological studies have shown that mucormycosis represents the third most encountered fungal infection after aspergillosis and candidiasis, in patients who underwent HSCT (6).

As previously noted, treatments options are limited, with radical surgery and high dose amphotericin B being still the most effective interventions (7). Amphotericin has been the cornerstone of treatment since its first use in 1957 (1), with great *in vitro* activity against fungi of the Mucorales group (8). Recent guidelines also support the use of azoles, such as isavuconazole and posaconazole, which are now considered viable therapeutic weapons either as primary treatments if there is a relative contraindication to amphotericin or for patients who are refractory or intolerant to amphotericin B (7, 9).

Through our study, we aimed to compare the current data with available obtained results, thereby providing a comprehensive assessment of the evolving landscape of diagnostic and therapeutic approaches of mucormycosis at our tertiary hospital of Liège. The purpose was to evaluate the efficacy and relevance of recommended diagnostic methods, and second to scrutinize the therapeutic regimens outlined in current guidelines. This study seeks to highlight potential gaps in clinical practice that may exist despite these recommendations.

## Materials and methods

### Study design, population and definitions

We conducted a retrospective monocentric cohort study in a tertiary care hospital (Centre Hospitalier Universitaire of Liège, Belgium). The study was conducted following the Declaration of Helsinki and approved by the local ethics committee of our hospital [Ref 2024/307-Comité d’Ethique Hospitalo-Facultaire Universitaire de Liège (707)].

Patients with mucormycosis, regardless of their age, and admitted between July 2018 and August 2023, were included. The diagnostic criteria for positive diagnosis of mucormycosis were based on the international consensus of the European Confederation of Medical Mycology and the Mycoses Study Group Education and Research Consortium (ECMM/MSG) (7).

Data were manually extracted from the electronic health records (EHR), including variables such as age, gender, comorbidities, treatment modalities, and clinical outcomes.

Exclusion criteria included patients who showed a false-positive polymerase chain reaction (PCR) coupled with a negative culture, and whose evolution was favorable despite any treatment.

Grading of the GVHD was performed by a senior hematologist in transplantation and was made according to international guidelines (10, 11).

### Biological and microbiological assessment

The analyzed samples came from broncho-alveolar lavage (BAL), solid tissue biopsy or sinus fluid. Fungal cultures were performed on Sabouraud agar medium incubated at 30 °C for a maximum of 30 days. PCR was performed by using the MucorGenius^®^ assay (PathoNostics, Netherlands) allowing a qualitative result.

Co-infections were distinguished from contaminations according to established criteria by the European Organization for Research and Treatment of Cancer/Mycosis Study Group Education and Research Consortium (EORTC/MSG).

Biological results were documented at the time of diagnosis within a margin of ±3 days.

Neutropenia referred to a neutrophil count below 500/mm^3^.

Our upper limit cut-off for ferritinemia was 275 μg/L.

### Imaging assessment

Imaging assessment for mucormycosis was conducted using chest Computed Tomography (CT), brain Magnetic Resonance Imaging (MRI), sinus CT or MRI and whole-body ^18^F-fluorodeoxyglucose ([^18^F] FDG) positron emission tomography (PET)/computed tomography (CT). All the exams were interpreted by a senior radiologist and nuclear physician.

For chest CT findings, indicators suggestive of pulmonary mucormycosis included the presence of nodules, the reversed halo sign, pleural effusion, ground-glass opacity, nodular consolidations, or alveolar infiltrates. Additionally, other chest CT features, such as the presence of a tracheal mass, were also considered (7, 12).

In the case of sinus involvement, CT findings suggestive of mycotic infections including mucormycosis, were opacification of the sinus cavities, tissue thickening associated with osteolytic components. MRI confirmed the necrotic nature of the lesions with a lack of enhancement of the mucosa in T1 with gadolinium. In T2, fungal elements appear classically in hyposignal with surrounding structures that may be in hypersignal (13).

### Follow-up assessments

According to the established response criteria by EORTC/MSG, Complete Response (CR) requires survival, resolution of all attributable symptoms, eradication of infection based on culture or other markers, as well as radiological clearance. Partial Response (PR) involves survival, improvement in physical findings, and reduction of radiological lesions of at least 25% at day 42 and 50% at day 84. Stable Response (SR) involves survival with no significant clinical or radiological improvement or progression of disease. Progressive Disease (PD) indicates worsening clinical symptoms, radiological deterioration, or evidence of new or expanding fungal sites despite treatment.

Biological follow-up was made on day 28, 42, and 84 within a margin of ±3 days.

Renal toxicity was assessed by serum creatinine levels.

Drug induced liver injury (DILI) was made based on Aspartate Transaminase (AST), Alanine Transaminase (ALT), Alkaline Phosphatase (ALP), International Normalized Ratio (INR), total bilirubin and Gamma-Glutamyl Transferase (GGT) and grading was set according to the developed scale of the Drug Induced Liver Injury Network (DILIN) (14).

The mortality rate was evaluated at day 84.

Concerning patients with HSCT, death was assessed reportedly to Copelan criteria (15).

### Statistical analysis

Quantitative variables were reported as frequencies, and continuous variables were summarized as medians along with their respective ranges to provide a comprehensive overview of the data distribution. We employed Student’s *t*-test to compare the means between the two groups.

To estimate the overall survival (OS) probabilities, the Kaplan-Meier method was employed.

The proportional hazards’ assumption, the impacts of outliers, and the linearity of the relationship between the log hazard and covariates were thoroughly examined. Results were deemed significant if the *p*-value was inferior to 0.05.

Statistical analysis was performed using RStudio (version 2024.09.1 + 394) and the R programming language (version 4.4.2). The Kaplan-Meier survival analysis was conducted using the “survival” and “suvminer” packages.

## Results

During the study period, 21 patients were detected positive for mucormycosis. According to our study inclusion criteria, 17 patients were enrolled (Supplementary Figure 1), with a median age of 57 years [IQR, 47–64], and 76% were male (Table 1). Hematological malignancies were the predominant underlying condition, affecting 14 patients (82%). Thirteen patients had a history of HSCT, one of them being performed for chronic granulomatous disease. Additionally, two patients (12%) had received a solid organ transplant (kidney transplantation).

**TABLE 1:** Review table of patients’ characteristics.

Parameter	All patients (*N* = 17)	Survivors (*N* = 6)	Non-survivors (*N* = 11)	*P*-value
Age (years)	57 (47–64)	37 (17–48)	64 (59–67)	**0.001**
Male patients (%)	13 (76%)	4 (67%)	9 (82%)	0.55
T15l**Underlying condition**				
Hematological malignancy (%)	14 (82%)	3 (50%)	11 (100%)	**0.02**
* Acute leukemia (%)	4 (24%)	1 (17%)	2 (18%)	–
* Lymphoma (%)	3 (18%)	1 (17%)	2 (18%)	–
* Aplastic anemia (%)	1 (6%)	1 (17%)	0 (0%)	–
* Multiple myeloma (%)	2 (12%)	0 (0%)	2 (18%)	–
* Other (%)	4 (24%)	0 (0%)	4 (36%)	–
HSCT (%)	13 (76%)	3 (50%)	10 (91%)	
Solid organ transplant (%)	2 (12%)	2 (33%)	0 (0%)	0.09
Diabetes (%)	4 (24%)	1 (17%)	3 (27%)	1.00
Neutropenia at diagnosis (%)	2 (12%)	0 (0%)	2 (18%)	0.52
Received corticosteroids in the previous 30 days (%)	11 (65%)	5 (83%)	6 (55%)	0.33
Received chemotherapy in the previous 30 days (%)	5 (29%)	2 (33%)	3 (27%)	1.00
Immunosuppressive drugs (%)	9 (53%)	3 (50%)	6 (55%)	1.00
* Tacrolimus (%)	6 (35%)	3 (50%)	3 (27%)	–
* MMF (%)	2 (12%)	2 (33%)	0 (0%)	–
* TKI (%)	2 (12%)	0 (0%)	2 (18%)	–
* Jak-inhibitor (%)	1 (6%)	0 (0%)	1 (9%)	–
Posaconazole prophylaxis (%)	3 (18%)	0 (0%)	3 (27%)	0.52
Voriconazole prophylaxis (%)	4 (24%)	2 (33%)	2 (18%)	0.58
Graft Versus Host Disease (%)	6 (35%)	1 (17%)	5 (45%)	0.33
* Acute GVHD (%)	3 (18%)	0 (0%)	3 (27%)	–
* Chronic GVHD (%)	3 (18%)	1 (17%)	2 (18%)	–
High ferritin level (%)	11/12 (92%)	5/5 (100%)	6/7 (86%)	–
Hemoglobin (g/dL)	8.7 (7.6–9.3)	8.9 (7.9–10)	8.6 (7.3–9.1)	–
Platelets (mm^3^)	35,000 (23,000–87,000)	30,500 (26,000–10,1750)	45,000 (23,000–67,500)	–
Neutrophils (mm^3^)	1,890 (1,250–3,280)	2,395 (1,320–3,577)	1,890 (685–3,260)	–
Lymphocytes (mm^3^)	450 (60–500)	595 (40–610)	455 (350–470)	–
CRP (mg/L)	129 (24–146)	21 (20–82)	135 (106–148)	–
Ferritin (μg/L)	3,062 (1,965–7,345)	6,642 (3,426–9,457)	1,972 (1,253–4,255)	**0.03**
AST (U/L)	25 (16–42)	29 (21–103)	25 (16–36)	–
ALT (U/L)	23 (10–44)	53 (26–217)	16 (9–27)	–
INR	1.13 (1.01–1.2)	1.07 (1.01–1.11)	1.165 (1.1–1.2)	–
Total bilirubin (μmol/L)	0.83 (0.45–1.17)	0.65 (0.45–0.90)	0.865 (0.7–1.5)	–
ALP (UI/L)	97 (72–172)	107 (87–112)	94 (71–179)	–
Creatinine (mg/dL)	1 (0.7–1.52)	1.14 (0.72–1.78)	1 (0.8–1.3)	–

Bold values indicate signicant statistically results (*p* < 0.05). AST: Aspartate Transaminase; ALT, Alanine Transaminase; ALP, Alkaline Phosphatase; AST, Aspartate Transaminase; CRP, C-reactive protein; GVHD, Graft Versus Host Disease; HSCT, hematopoietic stem cell transplant; INR, International Normalized Ratio; MMF, mycophenolate mofetil; TKI, tyrosine kinase inhibitors.

The median age of all patients included was 57 years, with a median of 37 years among survivors and 64 years among the non-survivors.

At the time of diagnosis, only two patients (12%) presented neutropenia. A sizable proportion, 63% (*n* = 11), had received corticosteroids within the 30 days preceding their diagnosis. Five patients (29%) had received chemotherapy during the 30 days prior to the diagnosis as part of the conditioning regimen for HSCT, and nine patients (53%) were on immunosuppressive therapy with calcineurin inhibitor (tacrolimus) being the most common treatment (67%).

Lymphopenia was also encountered in most patients with a median lymphocyte count of 450/mm^3^ [IQR, 60–500]. Among patients who survived, the median lymphocyte count was 595/m^3^ [IQR, 40–610], while patients who died had a median of 455/mm^3^ [IQR, 350–470].

Antifungal prophylaxis was administered in several cases: voriconazole 600 mg daily dose was used in four patients, evenly distributed across both categories, while posaconazole 300 mg was prescribed for three patients.

Among the study cohort, six patients (35%) had GVHD, and three of them being acute.

Biochemical analysis revealed that ferritin levels were measured in 12 of the 17 patients, with elevated levels detected in 11 cases (92%).

The diagnosis of mucormycosis was established using samples from broncho-alveolar lavage (BAL) (*n* = 15, 88%), solid tissue biopsy (*n* = 3, 18%) or sinus fluid (*n* = 1, 6%). PCR was performed for 16 patients, yielding positive results in all cases. Results were obtained approximately 4 days after sampling [IQR, 2–5] (Table 2). However, cultures were positive for a Mucorales agent in only three cases (18%) and molecular sequencing was conducted directly on tissue sample in four other cases (23%), both detecting *Rhizopus arrhizus* (*n* = 2), *Rhizopus microsporus* (*n* = 2), *Rhizopus pusillus* (*n* = 1) and *Lichteimia corymbifera* (*n* = 1).

**TABLE 2:** Review table of diagnostic methods.

Category	Characteristics
Infection status	Proven: 4 (24%)
	Probable: 4 (24%)
	Possible: 9 (52%)
Tested sample	BAL: 15 (88%)
	Solid organ biopsy: 3 (18%)
	Sinuses fluid: 1 (6%)
Pan-Mucorales PCR	16/16 (100%)
Number of days before first positive PCR	4 (2–5)
Positive culture for Mucorales	3 (18%)
Molecular sequencing confirming Mucorales identification	4 (23%)
Non-septate rubanned hyphae on histology	6 (35%)
Mucorales species	*Rhizopus arrhizus*: 2
	*Rhizopus microsporus*: 2
	*Rhizopus pusillus*: 1
	*Lichteimia corymbifera*: 1
Dual infection	Bacterial: 2 (12%)
	Viral: 0
	Fungal: 7 (41%)
Clinical presentation	Localized: 16 (94%)
	Disseminated: 1 (6%)
Affected organ	Lungs: 16 (94%)
	Sinuses: 1 (6%)
	Brain: 1 (6%)
Radiological pulmonary aspect	Nodules: 8 (50%)
	Reversed Halo sign: 2 (12%)
	Pleural effusion: 4 (25%)
	Ground glass opacity: 8 (50%)
	Alveolar condensation: 15 (94%)
PET/CT at diagnosis	4 (23%)

PCR, polymerase chain reaction; PET/CT, positron emission tomography/computed tomography.

Direct histological examination performed on BAL samples or tissue biopsy revealed hyphae compatible with mucormycosis in six cases (35%).

Mucormycosis was presented as a localized infection in 16 patients (94%) and as a disseminated form once. Pulmonary involvement was observed in 16 patients (94%), with brain dissemination occurring in one instance; sinus involvement was noted in one case.

Co-infections were identified in 8 patients. Among these, six had fungal co-infection (41%), including five probable *Aspergillus* infections— *A. fumigatus (n* = 2), *A. flavus* (*n* = 1) and *A. niger* (*n* = 1)— one possible case of *Candida glabrata was identified*, and four patients (57%) were still alive on day 84. Additionally, bacteremia with *Enterococcus faecium* occurred twice. Based on SARS-CoV-2 PCR performed on BAL fluid, no evidence of concomitant COVID-19 was detected.

On chest-CT scans, the most common finding was alveolar condensation, seen in 94% of pulmonary cases. Additionally, nodules and ground-glass opacities were described in 50% of the patients. “Reversed halo” was observed in two patients (12%) (Supplementary Figure 2).

[^18^F] FDG PET/CT was performed in four patients for the initial evaluation of mucormycosis infection and has shown moderate to intense hypermetabolic activity of the described CT lesions.

In our study cohort, one patient declined treatment and opted for palliative care.

Surgery including lobectomy and functional endoscopic sinus surgery (FESS) was performed in four cases, three of whom are on the survivors’ list. One patient, listed in the non-survivors’ column had benefited from a lobectomy, even if he reached partial response and survived 210 days, he went into palliative care due to his hematological malignancy and died. Surgery couldn’t be performed for the remaining patients (Table 3).

**TABLE 3:** Review table of treatment approaches and follow-up.

Parameter	All patients (*N* = 17)	Survivors (*N* = 6)	Non-survivors (*N* = 11)
Surgery	4 (24%)	3 (50%)	1 (9%)
First-line monotherapy	13 (76%)	5 (83%)	8 (72%)
* L-AmB < 5 mg/kg/day (%)	1 (8%)	–	1 (9%)
* L-AmB 5 mg/kg/day (%)	8 (61%)	4 (80%)	4 (50%)
* L-AmB 10 mg/kg/day (%)	2 (16%)	–	2 (25%)
* Isavuconazole 200 mg/day (%)	2 (16%)	1 (20%)	1 (9%)
* Therapeutic escalation to bitherapy (%)	4 (30%)	2 (34%)	2 (18%)
Decrease in L-AmB dosage or switch to azole for toxicity reason (%)	2 (12%)	–	2 (18%)
First-line bitherapy (Azole + L-AmB) (%)	3 (17%)	1 (17%)	2 (18%)
Secondary prophylaxis (%)	9 (53%)	5 (83%)	4 (36%)
Developed treatment-related toxicity (%)	8 (47%)	2 (33%)	6 (55%)
Favorable radiological evolution (%)	8 (47%)	6 (100%)	3 (27%)
PET/CT for follow-up (%)	7 (41%)	3 (50%)	4 (36%)
Favorable PET/CT for follow-up (%)	5/7 (71%)	3/3 (100%)	2/4 (50%)
CR (%)	8 (47%)	6 (100%)	2 (18%)
PR (%)	1 (6%)	–	1 (9%)
SD (%)	2 (12%)	–	2 (18%)
PD (%)	6 (35%)	–	6 (55%)
Total number of days of antifungal therapy	–	378 (181–475)	99 (11–178)
Creatinine at day 28 (mg/l)	1.83 (1.39–2.75)	1.61 (1.41–4.38)	2.04 (1.33–2.15)
Creatinine at day 42 (mg/l)	1.69 (1.36–2.15)	2.12 (1.28–3.07)	1.65 (1.34–1.79)
Creatinine at day 84 (mg/l)	1.3 (0.99–2.9)	2.78 (0.76–6.19)	1.09 (0.77–2.07)
DILI at day 28 (%)	3 (18%)	2 (33%)	1 (9%)
DILI at day 42 (%)	3 (18%)	1 (17%)	2 (18%)
DILI at day 84 (%)	3 (18%)	1 (17%)	2 (18%)
Cause of death (%)			
* Mucormycosis (%)	4 (24%)	–	4 (36%)
* GVHD (%)	2 (12%)	–	2 (18%)
* Other (%)	4 (24%)	–	4 (36%)
Life-end decision (%)	2 (12%)	–	2 (18%)

CR, Complete Response; DILI, drug liver induced injury; GVHD, Graft Versus Host Disease; L-AmB, Liposomal Amphotericin B; PD, Progressive Disease; PET/CT, positron emission tomography/computed tomography; PR, Partial Response; SD, stable disease.

Monotherapy was proposed as the first-line treatment in 76% of cases (*n* = 13), with Liposomal Amphotericin B (L-AmB) being the most used agent (*n* = 11, 85%). Among these 13 patients, 11 were treated with L-AmB: eight received a dose of 5 mg/kg/day, two received 10 mg/kg/day, and one received less than 5 mg/kg/day. Due to their impaired renal function, the remaining two patients were treated with isavuconazole with an induction phase dosage of 200 mg/8 h for 2 days, then 200 mg/day for maintenance. Both patients achieved resolution of their infection.

Two patients (12%) developed L-AmB-associated toxicity, requiring a switch to azoles or a decreased posology; however, they didn’t survive.

Therapeutic escalation to combination therapy associating L-AmB with isavuconazole or posaconazole was performed in four patients (30%), two of whom belonged to the non-survivors’ group.

[^18^F] FDG PET/CT was used as a tool for follow-up in 41% of patients (*n* = 7), being performed after a median duration of 3.5 months [IQR, 3–4], with favorable outcomes reported in five cases.

**FIGURE 1:**
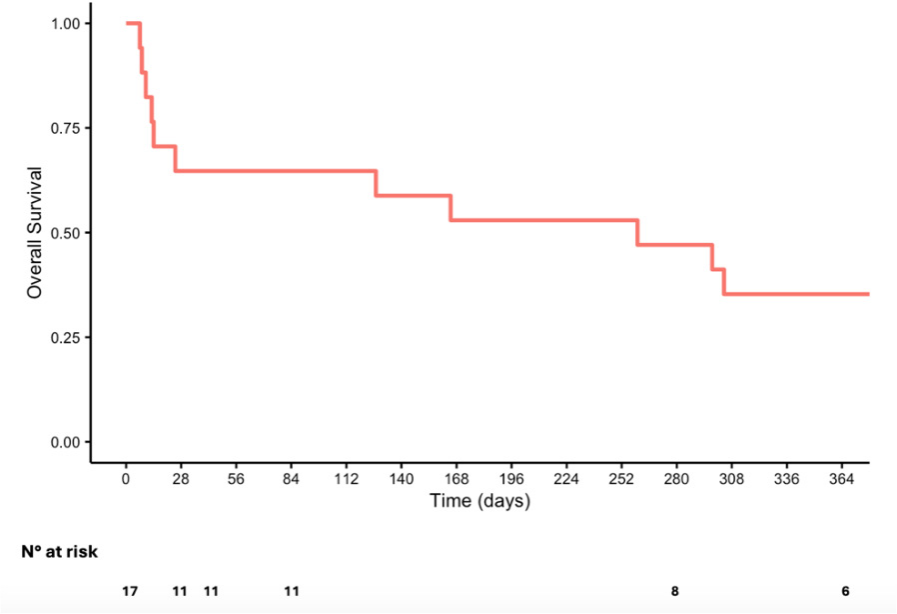
Kaplan-Meier curve of overall survival.

The overall survival data for patients included in this study is illustrated in Figure 1. The mortality rate reached 35% on day 28 and remained the same for day 42 and 84.

**FIGURE 2:**
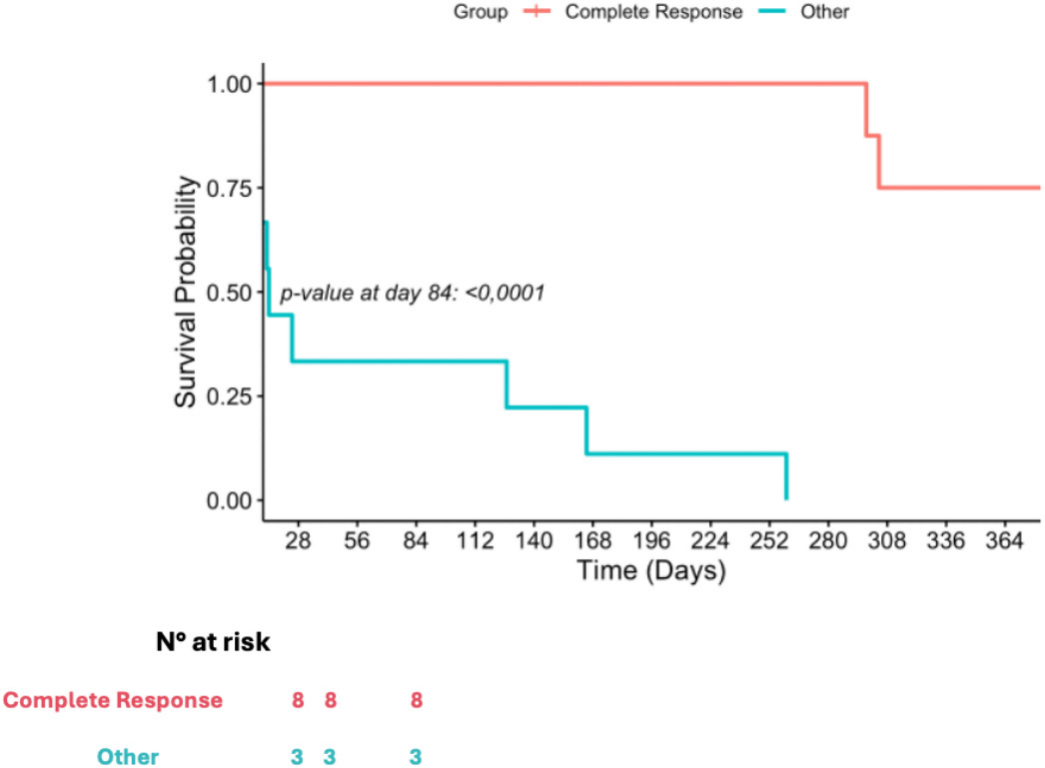
Kaplan-Meier curve of according to treatment response.

After 1 year of follow up, we recorded 25% mortality among patients who had a complete response to their infection, while on the other side, mortality reached 100% with a median survival time of 14 days [IQR, 9–146]. A comparative in OS between patients who had a CR and those who did not, is illustrated in Figure 2.

**FIGURE 3:**
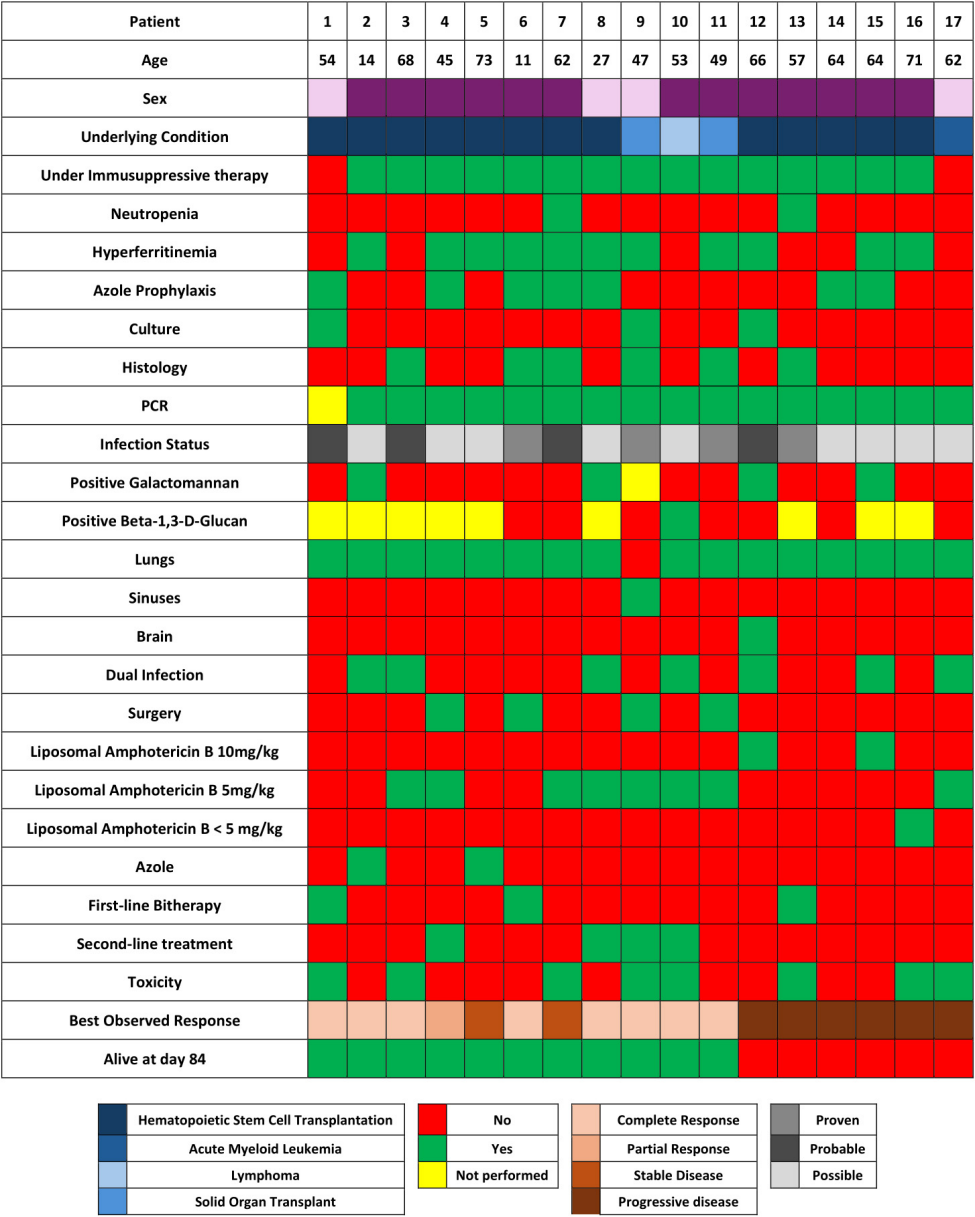
Comprehensive review table of patients’ characteristics.

Figure 3 provides a comprehensive overview of all patients’ characteristics discussed in this study.

According to Copelan’s criteria, GVHD was identified as the cause of death in 18% of cases (*n* = 2), while mucormycosis was the primary cause of death in 36% of cases (*n* = 4). No significant difference in mortality was observed among patients who developed GVHD.

Other causes of death were malignant hypercalcemia, bacterial pneumonia and subsequent COVID-19 infection.

## Discussion

Our monocentric study is the second retrospective study providing insights on mucormycosis in a Belgian University Hospital since the study made by Saegeman et al. (3, 5). Our results add to the understanding of this complex high-mortality infection. Mucormycosis remains a significant cause of high mortality in patients suffering from a hematological malignancy, reaching a rate of 61% despite recent advances in therapy (2). Another retrospective study conducted by Rothe and al. in the University Hospital of Berlin reviewing cases from 2016 to 2019 reported a mortality rate of 100% among critically ill patients with pulmonary mucormycosis, highlighting the severe outcomes associated with this infection (16).

In line with earlier literature, our study confirms hematological malignancies, hyperferritinemia, GVHD, and immunosuppressive therapy as primary risk factors. Interestingly, while neutropenia is commonly cited as a major risk factor for mucormycosis (17), it was present in only 12% of our cases. This suggests that the underlying susceptibility may stem from qualitative factors related to immune response rather than merely quantitative ones; this could be due to the large use of immunosuppressive drugs in patients who benefited from a stem cell or solid organ transplant. However, unlike our expectations, no significant difference in survival probability was registered in mortality in the group of patients who had GVHD. Another explanation may be the longer exposition to treatment for hematological patients due to new treatment for relapsing disease. But recent data from France still showed a strong association between mortality and neutropenia (18). Besides, the younger patients of our study demonstrated a better survival rate, suggesting that age was another determining factor.

Additionally, immune dysfunction is central to invasive mucormycosis. Beyond quantitative lymphocyte depletion, Potenza and al. showed that Mucorales-specific T cells can be polarized toward a Th2-type cytokine profile, notably interleukin-4 or interleukin-10 production, which was associated with proven invasive mucormycosis in hematologic patients; highlighting that qualitative and pathogen-specific immunity may matter as much as absolute counts (19). Also, in COVID-19-associated mucormycosis, lymphopenia and broader immune dysregulation were also frequently reported as contributing factors to susceptibility (20). In our cohort, lymphopenia was frequent and profound, yet lymphocyte count did not differ significantly between survivors and non-survivors, likely reflecting uniformly severe immunosuppression and limiting the prognostic value of lymphocyte count alone.

A study conducted by Lionakis et al. (19), has studied the breakthrough of invasive mold infections in patients receiving azole prevention. While these agents are intended to prevent invasive fungal infections (IFIs), their use can inadvertently create selective pressure, leading to the development of resistant fungal species, including mucormycosis (21). In our study, 7 of the 17 patients had prior antifungal prophylaxis, which made the management of their cases complicated, as only three of them had a complete response.

Diagnosing mucormycosis infection is still challenging, as the challenges are compounded by the limited sensitivity and specificity of traditional methods. Imaging, for instance, is primordial in case of a suspected pulmonary mucormycosis, the modality of choice being CT scan as some findings like the presence of a reverse halo is highly suggestive (7), unfortunately it is seen in late stages of the disease as the fungal infection progresses (12). Sinonasal imaging and 0.

The EORTC/MSG categorized the definitions of invasive fungal disease (IFD) into three main levels, based on diagnostic certainty. Proven IFD requires detection of fungal elements in diseased tissue through histology or culture from sterile sites, regardless of clinical symptoms or host factors. Probable and possible IFD both involve host factors such as immunosuppression, and clinical evidence; the main difference relies on mycological evidence based on results from direct or indirect testing like antigen testing (22). Actual guidelines from the European Confederation of Medical Mycology (ECMM) strongly suggest realizing histopathological examination as it is still the gold standard for the diagnosis of mucormycosis. Microbiological culture on a solid tissue biopsy from a bronchoscopic biopsy; percutaneous needle biopsy or surgical resection can also confirm the diagnosis. However, earlier studies have shown that microbiological cultures are often limited by poor sensitivity as rates can be as low as 20%–30% of the cases (7, 17). In another cohort study grouping 319 patients, culture was positive in only 3.1% of the cases (23), in our study, culture was positive in 18% of the cases.

According to these definitions, only 24% of infections in our study cohort were classified as proven. Incorporating positive PCR into diagnostic criteria could potentially reduce the risk of mismanagement in these patients, particularly given the challenges of obtaining sterile site samples in critically ill and immunocompromised population.

Unlike traditional culture methods, which require viable organisms and can take days to weeks to yield results, PCR can provide results within a few hours; this rapid turnaround time is especially critical in the context of mucormycosis, where prompt diagnosis and initiation of antifungal treatment significantly change patient survival (24, 25).

In addition to being difficult to treat, timely and accurate diagnosis of mucormycosis is crucial, as mortality rate can exceed 50% if the right treatment dosage is delayed by 6 days or more (26), therefore, these traditional diagnostic methods often fail to provide the quickest and the most optimal therapeutic strategy. Molecular techniques have emerged as vital tools for the rapid and accurate diagnosis of mucormycosis. These techniques, which include polymerase chain reaction (PCR), quantitative PCR (qPCR), multiplex PCR assays, and high-resolution melt analysis assay (HRMA), allow for the detection of specific fungal Deoxyribonucleic Acid (DNA) sequences in clinical specimens, significantly enhancing diagnostic sensitivity and specificity. One of the primary advantages of PCR-based methods is their ability to identify Mucorales directly from clinical samples, including plasma/serum, tissue, respiratory secretions, and even urine. These PCR assays target different sequences, including ribosomal 18S, 28S and Internal Transcribed Spaces (ITS) regions, the FTR1 gene, or DNA encoding CotH, a spore-coating encoding protein specific to Mucorales which has so far been described in a murine model (27, 28).

The use of PCR on blood samples is a non-invasive approach which provides early detection and can be used as a screening method in at-risk patients (2). It is unfortunately not available yet in our hospital laboratory.

Quantitative PCR has shown a particular promise in the diagnosis of mucormycosis. By amplifying specific DNA sequences associated with Mucorales, qPCR not only detects the presence of the pathogen but also quantifies the fungal burden essentially in blood samples, facing less quality or dilution issues compared to LBA. This quantitative approach can help clinicians assess the severity of the infection and monitor treatment efficacy (29). Our hypothesis is that qPCR can be valuable in post-surgical patients, as it could allow infection monitoring and hence treatment adjustments.

Multiplex PCR assays, such as MucorGenius^®^, which are used by our microbiological laboratory, allow for the simultaneous detection of the most frequent Mucorales agents. These assays can help differentiate between mucormycosis and other invasive fungal infections, thereby guiding proper antifungal therapy. The MucorGenius^®^ assay has shown high sensitivity and specificity, detecting Mucorales DNA in clinical specimens that would have otherwise gone undiagnosed (23).

Recent advances in mucormycosis diagnostics, particularly molecular techniques such as PCR and multiplex assays, have significantly improved the speed and accuracy of detection. Millon’s study highlights how an increasing number of hospitals are integrating this tool into their workflows (30). Additionally, evidence from a recent meta-analysis supports the use of molecular methods and their integration in updated diagnostic guidelines (31). However, the sensitivity and specificity of PCR can vary significantly based on differences in protocols, sample types, untargeted DNA regions, leading to discrepancies in diagnostic accuracy. The absence of circulating fungal antigens to confirm PCR findings further complicates result interpretation, particularly in cases with high cycle threshold (Ct) values, which may indicate either low fungal burden or contamination.

Contamination is also another critical issue, as even traces amount of fungal DNA in reagent or laboratory environments can lead to false positives. This challenge is especially relevant for ubiquitous fungi like Mucorales, emphasizing the need for stringent quality control measures to reduce diagnostic errors (32).

ITS sequencing can also be used for the detection of fungi in tissue; however, it has lower sensitivity compared to targeted PCR, therefore we prefer to use *Aspergillus* PCR or Mucorales PCR in order to increase the diagnostic sensitivity. At CHU of Liège, we perform ITS sequencing to detect fungi (33) directly on tissue or biological fluids (except blood) when targeted PCRs are negative. Recently, we introduced Mucorales qPCR in blood, providing a non-invasive screening method for at-risk patients. Unfortunately, this approach shows lower sensitivity for *Aspergillus* detection in blood. Therefore, another promising technique could be the use of metagenomic Next-Generation Sequencing (mNGS), allowing the detection of a broad panel of fungi. Some authors have successfully used mNGS in the diagnosis of mucormycoses in blood (34). Others have applied mNGS for the detection of invasive mycoses in hematopoietic stem cell transplant recipients (35). In this study conventional microbiological tools (CMT) including qPCR and mNGS were compared. The results showed that mNGS was much more sensitive than CMT. Furthermore, mNGS enabled modification of the empirical treatment and improved patient outcomes, demonstrating its added value.

Co-infections of mucormycosis and aspergillosis have increasingly been recognized in immunocompromised patients. This dual infection poses significant diagnostic and treatment challenges due to overlapping clinical presentations. Studies have revealed varying prevalence rates, suggesting that co-infections are not uncommon but may often go undetected due to limitations in diagnostic methodologies (30). In a study gathering 67 cases, ran by Sang Hyun Ra and al, it was noted that approximately 31% patients with proven mucormycosis had aspergillosis detected through PCR and culture methods (36), which is a rate similar to our results as 35% of the patients had aspergillosis. Another report by Aerts et al. (37) has given further evidence from serum Mucorales PCR studies in hematology patients, as it underscores the challenge of identifying these co-infections promptly. For instance, qPCR. has demonstrated a higher detection in patients with invasive aspergillosis, suggesting that the incidence of co-infections may go unrecognized without performing fungal PCR screening (37). Surprisingly, in both studies, mortality wasn’t significantly higher among patients who presented the dual infection. This could be explained by the fact that we may be “overtreating” these patients regarding their co-infection with *Aspergillus spp*. Given these points, we should nevertheless keep particular attention in patients with a confirmed invasive aspergillosis who don’t improve after starting treatment.

Current guidelines for the treatment of mucormycosis emphasize rapid diagnosis and multimodal approach combining antifungals, surgery and management of underlying risk factors. The European Confederation of Medical Mycology (ECMM) and Mycoses Study Group recommend high-dose L-AmB as the first line therapy, administered at 5–10 mg/kg daily, with higher doses for cases involving CNS (7, 38). This is bolstered by the study ran by the Prospective Antifungal Therapy Alliance (PATH), showing that early administration of L-AmB significantly improves survival outcomes, with a reported 12-week survival probability rate at 0.72 when started within 3 days of diagnosis (39), however their overall survival was estimated at 41%.

Only few prospective studies involving patients infected with mucormycosis have been published. The first was the DEFEAT Mucor trial (40), as it evaluated the combination of Deferasirox and L-AmB initially supported by studies on murine model (41). Unfortunately, an excessive mortality rate was seen in the case-control group. In another study that evaluated the efficacy and safety of use of a high dose regimen of L-AmB at 10 mg/Kg, mortality rate at week 12 was 38% with 32% of complete response (42). The last published study was the VITAL trial that compared the use of Isavuconazole to L-AmB, as it has shown no significant improvement in overall survival, all-cause mortality rate reaching 43% after 84 days (9). In the present study, we recorded a mortality rate of 35% on day 28, 42, and 84, as well as the median survival time of 14 days [IQR, 9–146] for patients who didn’t reach CR, suggesting that early diagnosis and management are decisive for maximizing patients’ chances of survival. These findings strengthen the case of implementing PCR as a diagnostic tool.

While current guidelines and other retrospective studies don’t support the use of dual therapy as it is associated to poorer outcome (7, 43), in a report from the SEIFEM (Sorveglianza Epidemiologica Infezioni Fungine Emopatie Maligne) and FUNGISCOPE registries, combined antifungal therapy was used as a salvage therapy in 29 patients after receiving monotherapy for a median duration of 18 days, and was associated with 34% of complete responses after (44).

Our data has registered three patients who benefited from the combination of L-AmB with posaconazole or isavuconazole as a first line therapy and four others who had a therapeutic escalation due to progressive disease. Among this group of seven patients, the survival rate at 84 days reached 72%, suggesting that a reconsideration of dual therapy may be warranted for certain patients. Patients who are not allowed to undergo surgery may be good candidates for bitherapy in case of bad initial response.

Assessing infected patients with mucormycosis is challenging, actual guidelines suggest performing weekly CT imaging, to distinguish from a partial response, stable or a progressive disease; in our study cohort, radiological assessment wasn’t performed on a weekly basis, as we monitored both clinical and biological evolution. Millon and al, have suggested a microbiological approach with the use of qPCR for monitoring the infection. However, no study to date has discussed its potential efficacy (29, 45).

[^18^F] FDG PET/CT was also one of our used tools for follow-up as it was performed in seven of our patients and contributed to a therapeutic escalation to bitherapy in two cases (Supplementary Figure 3). This approach compared to conventional imaging in fungal diseases could lead to a faster detection of infected sites in one whole-body study and a better therapeutic efficacy monitoring based on metabolic activity (46, 47). However, precise definitions are yet to be established, and further prospective studies are necessary to standardize its use.

Treatment approaches for mucormycosis also suffer from lack of standardization. Although guidelines exist, there is significant variability in the dosing, sequencing of antifungal agents, and the use of adjunct therapies like surgery. This inconsistency stems from the rarity of mucormycosis, which limits large-scale studies, and the urgency of treating a rapidly progressing infection. Consequently, treatment decisions often depend on local practices, drug availability, and most importantly patients’ factors, all contributing to differences in clinical outcomes across settings. Standardized treatment protocols are essential to improve survival rates and ensure consistent care.

Evaluating antifungal resistance in mucormycosis presents one of the other significant challenges due to the limited availability of standardized microbiological methods. Traditionally, antifungal susceptibility testing for Mucorales has been hindered by the nature of these fungi and the lack of standardized protocols and guidelines for MIC interpretation. Traditional culture-based techniques remain the most common approach for assessing antifungal resistance, but are often unreliable, failing to provide results in a timely manner, while mucormycosis expands quickly. Resistance mechanisms are complex and involve genetic factors, such as genome duplications, and environmental factors that can affect drug efficacy, and molecular techniques have yet proven their application in this matter, as it is still under investigation (48).

This study is subject to several limitations. Being a single-center study, the findings may not be generalizable to other healthcare settings. The small sample size reduces the statistical power and subgroup analysis. Additionally, reliance on retrospective data may have introduced biases, including incomplete or inconsistent record-keeping. The absence of standardized follow-up protocols also limits insights into long-term outcomes.

In conclusion, we managed to reach a 12-week survival rate of 65%, which is slightly lower than current literature. We demonstrated a good survival at 1-year (75% OS) for patients who reached a CR, outlining the importance of early aggressive and multidisciplinary management. We highlighted the key challenges associated with diagnosing and managing mucormycosis, a rare but severe infection. Early diagnosis, tailored antifungal regimens, and surgical interventions, when feasible, remain key to improving patient outcomes. Future studies should focus on redefining diagnostic criteria by including PCR techniques and implementing the use of [^18^F] FDG PET/CT for follow-up. However, despite these advancements, the high mortality rate underscores the need for new agents; in the meantime, we emphasize the importance of surgery, high dose L-AmB and the adherence to actual guidelines, ensuring weekly CT for follow-up.

## Data Availability

The original contributions presented in this study are included in this article/Supplementary material, further inquiries can be directed to the corresponding author.
