# Supplementary figures and images for: Novel diagnostic approaches and therapeutic management of mucormycosis: insights from a retrospective monocentric cohort study
Source: Front Med (Lausanne). 2026 Feb 4;13:1715663. doi: 10.3389/fmed.2026.1715663 (PMC12913173; doi:10.3389/fmed.2026.1715663)

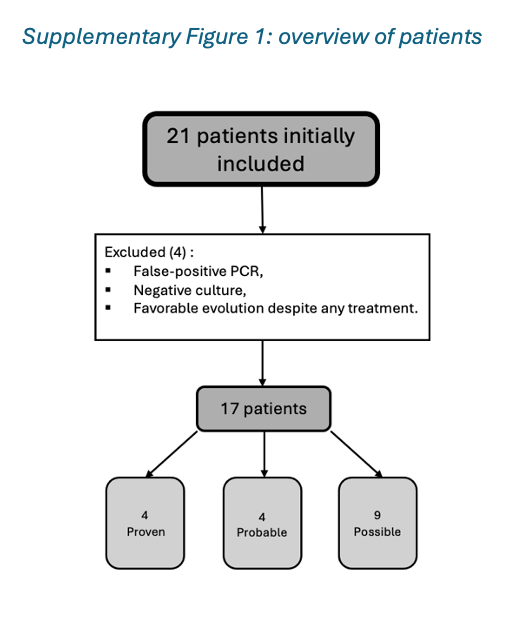

Supplement: Supplementary file 1 [file Image_1.tiff]

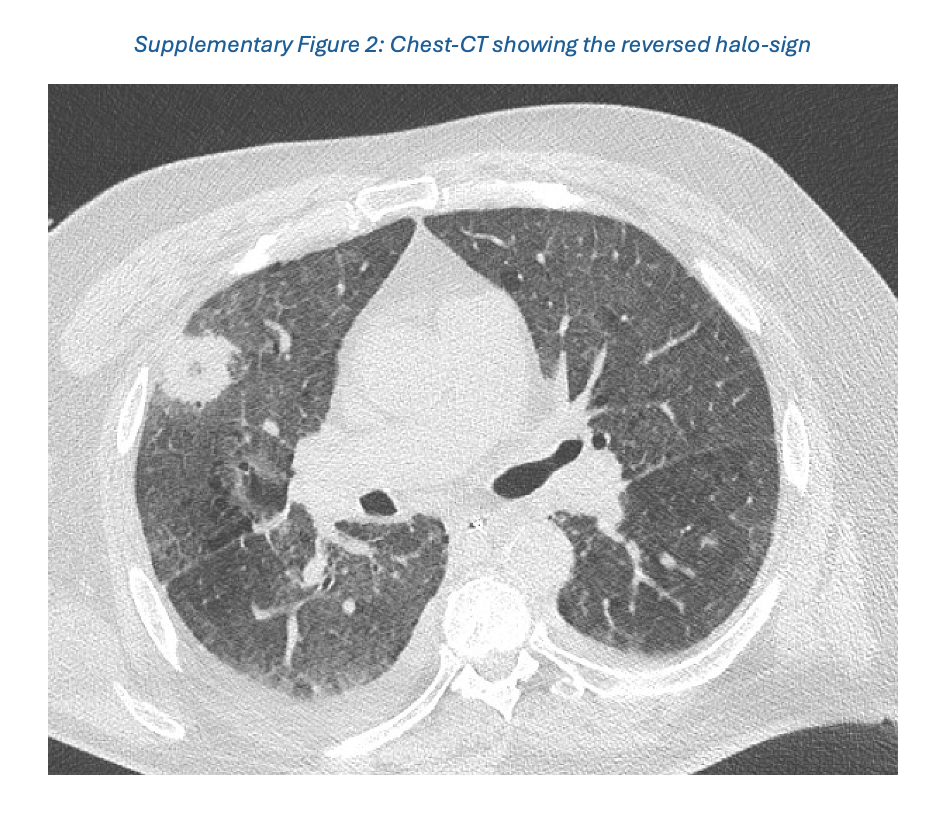

Supplement: Supplementary file 2 [file Image_2.tiff]

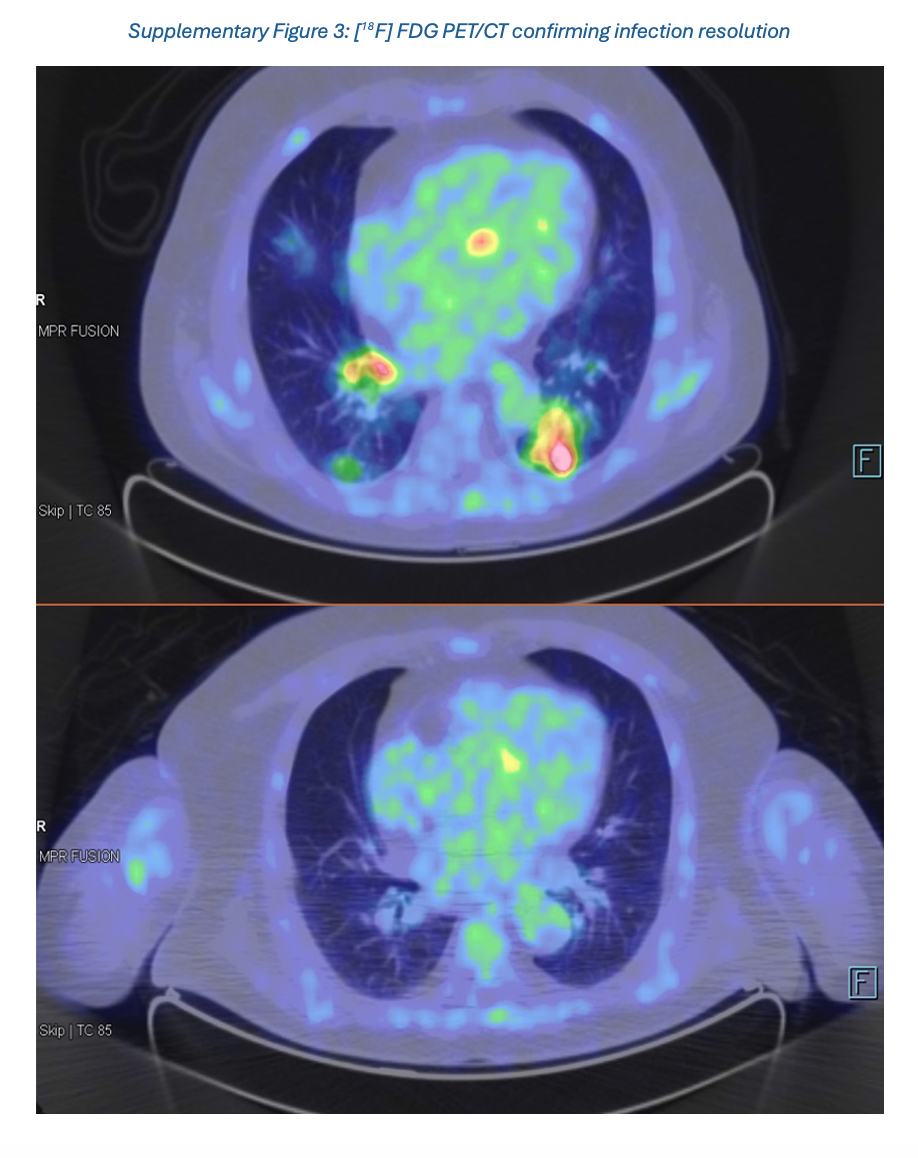

Supplement: Supplementary file 3 [file Image_3.tiff]
